# Visualization of hospital cleanliness in three Japanese hospitals with a tendency toward long-term care

**DOI:** 10.1186/1756-0500-7-121

**Published:** 2014-03-04

**Authors:** Reina Watanabe, Tomoko Shimoda, Rika Yano, Yasuhiro Hayashi, Shinji Nakamura, Junji Matsuo, Hiroyuki Yamaguchi

**Affiliations:** 1 Departments of Fundamental Nursing, Faculty of Health Sciences, Hokkaido University, North-12, West-5, Kita-ku, Sapporo 060-0812, Japan; 2 Department of Medical Laboratory Science, Faculty of Health Sciences, Hokkaido University, Nishi-5 Kita-12 Jo, Kita-ku, Sapporo, Hokkaido 060-0812, Japan; 3 Hokkaido University Hospital, Nishi-5 Kita-14 Jo, Kita-ku, Sapporo, Hokkaido 068-8648, Japan; 4 Division of Biomedical Imaging Research, Juntendo University Graduate School of Medicine, 2-1-1 Hongo, Bunkyo-ku, Tokyo 113-8421, Japan

**Keywords:** Hospital cleanliness, ATP bioluminescence, Stamp agar culture method, Long-term care, Japan

## Abstract

**Background:**

Hospital cleanliness in hospitals with a tendency toward long-term care in Japan remains unevaluated. We therefore visualized hospital cleanliness in Japan over a 2-month period by two distinct popular methods: ATP bioluminescence (ATP method) and the standard stamp agar method (stamp method).

**Methods:**

The surfaces of 752 sites within nurse and patient areas in three hospitals located in a central area of Sapporo, Japan were evaluated by the ATP and stamp methods, and each surface was sampled 8 times in 2 months. These areas were located in different ward units (Internal Medicine, Surgery, and Obstetrics and Gynecology). Detection limits for the ATP and stamp methods were determined by spike experiments with a diluted bacterial solution and a wipe test on student tables not in use during winter vacation, respectively. Values were expressed as the fold change over the detection limit, and a sample with a value higher than the detection limit by either method was defined as positive.

**Results:**

The detection limits were determined to be 127 relative light units (RLU) per 100 cm^2^ for the ATP method and 5.3 colony-forming units (CFU) per 10 cm^2^ for the stamp method. The positive frequency of the ATP and stamp methods was 59.8% (450/752) and 47.7% (359/752), respectively, although no significant difference in the positive frequency among the hospitals was seen. Both methods revealed the presence of a wide range of organic contamination spread via hand touching, including microbial contamination, with a preponderance on the entrance floor and in patient rooms. Interestingly, the data of both methods indicated considerable variability regardless of daily visual assessment with usual wiping, and positive surfaces were irregularly seen. Nurse areas were relatively cleaner than patient areas. Finally, there was no significant correlation between the number of patients or medical personnel in the hospital and organic or microbiological contamination.

**Conclusions:**

Ongoing daily hospital cleanliness is not sufficient in Japanese hospitals with a tendency toward long-term care.

## Background

Much attention has been focused on hospital-acquired infections in the last decade [1-3]. These infections can be acquired from microbe-contaminated hospital environments that are frequently touched by hands, namely “high-touch surfaces,” including doorknobs, guardrails in corridors, and overbed tables of inpatients [1]. Such sites are thought to provide the greatest risk for patients [1]. Therefore, efforts to improve hand hygiene and isolation practices have been implemented to help mitigate this problem on a worldwide scale, particularly in developed countries [1]. In fact, recent studies have shown that routine cleaning practices in hospitals are associated with a decrease in transmission of vancomycin-resistant *Enterococcus* or methicillin-resistant *Staphylococcus aureus*[4-7], suggesting a significant implication of hospital cleanliness in the control of hospital-acquired infections.

According to this concept of hospital cleanliness, recommendations and standards to improve hospital cleanliness have dramatically evolved. Several guidelines [8-10], such as those of the Centers for Disease Control and Prevention [10], strongly insist on the need for infection prevention and control programs, including appropriate monitoring of medical staff and housekeeping activities related to hospital cleanliness, to control hospital-acquired infection and predict the risk of patient infection. These guidelines similarly recommended cleaning and disinfection of surfaces in close proximity to the patient and those that are likely to be touched by the patient and medical staff members or housekeepers, although visual assessment of hospital cleanliness is still popular and has been believed to be linked with reduction of infection. In addition, both adenosine triphosphate (ATP) bioluminescence (ATP method) as indicators of general organic contamination [11-15] and the standard stamp agar method (stamp method) for monitoring microbiological contamination [16-18] have been available for monitoring hospital cleanliness.

Compared with other developed countries such the United States and countries in Europe, the average number of bed disability days in Japan hospitals is at least twice as high (approximate average, 19 days) as indicated by OECD health data [19]. This indicates that Japan hospitals have a tendency toward long-term care and evoke strong caution regarding hospital-acquired infections. Thus, the concept of hospital cleanliness to control hospital-acquired infection is well understood in Japan and other countries. However, in Japanese hospitals, daily hospital cleanliness has been limited to visual assessment with wiping, ignoring the hospital characteristic of long-term care, and more importantly has not been sufficiently supported by evidence-based studies on monitoring hospital cleanliness with a large amount of data.

In the present study, to define the actual conditions of cleanliness in Japan hospitals with a tendency toward long-term care, we attempted to visualize hospital cleanliness by testing 752 surfaces in three hospitals with both the ATP and stamp methods over a 2-month period.

## Methods

### Experimental design

**Figure 1:**
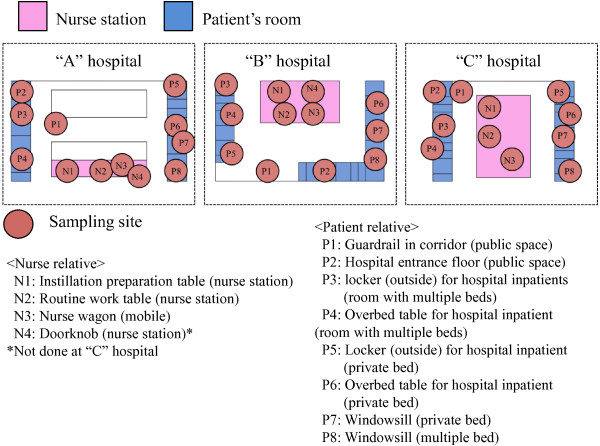
**Ward floor maps and sampling places.** The study was conducted at three differently sized hospitals (“**A**” hospital, “**B**” hospital, and “**C**” hospital) located in a central area of Sapporo, Japan. See the Methods. Each sampling site was sampled eight times in 2 months. N1-4, Nurse relative. P1-8, Patient relative.

This study was conducted at three hospitals of different sizes [“A” hospital (>500 beds), “B” hospital (100–500 beds), and “C” hospital (<100 beds)] located in a central area of Sapporo, Japan with the following sampling periods: “A” hospital, October 2011 to December 2011; “B” hospital, December 2011 to February 2012; and “C” hospital, November 2011 to December 2011. We also tested various nursing areas [N1, instillation preparation table (nurse station); N2, routine worktable (nurse station); N3, nurse wagon (mobile station in nurse area); N4, doorknob (nurse station)] and patient areas [P1, guardrail in corridor (public space); P2, hospital entrance floor (public space); P3, locker (outside) for hospital inpatients (room with multiple beds); P4, overbed table (room with multiple beds); P5, locker (outside) for hospital inpatient (room with private bed); P6, overbed table for hospital inpatient (room with private bed); P7, windowsill (room with private bed); P8, windowsill (room with multiple beds)] on three different ward units (Internal Medicine, Surgery, and Obstetrics and Gynecology) in each hospital (Figure 1). An attempt was made to also collect swab samples from places with the potential to mediate hospital-acquired infections, but insufficient areas and shapes too complicated for sampling were omitted from the sampled places. After initial testing, we conducted follow-up monitoring, sampling each 8 times in 2 months. Hospital cleanliness was evaluated using the ATP and stamp methods (see below for details). The total number of surfaces assessed was 752. Sampling of areas touched by nurses and patient relatives was regularly and simultaneously performed at around 11:00 am after cleaning in almost all cases, because general cleaning in these hospitals was usually performed from 8:00 am to 11:00 am. In addition, there were no inaccessible rooms during our study because the sampling places were limited and did not include clean rooms or patient treatment rooms.

### Assessment of the number of medical personnel and hospital inpatients

During the experimental period, we recorded the number of medical personnel (nurses and nurse aids) and hospital inpatients in each of the wards at the time of sample collection. No significant change in the number of patients or medical staff personnel (nurses, medical doctors, or medical assistants) per day among the wards was found in any of the hospitals. The total average number of patients and medical staff personnel per day in each hospital during the study period was 61.3 ± 9.9 (“A” hospital), 48.6 ± 6 (“B” hospital), and 30.7 ± 4 (“C” hospital), although these numbers changed depending upon the hospital size.

### Visual assessment and wiping

All of the hospitals had an ongoing program for hospital cleanliness comprising visual assessment according to a checklist (visual dirt, rubbish, smears, dust, grease, blood, fingerprints, and clinical waste on clinical surfaces) as previously described [20] and wiping with detergents such as Magiclean (Kao, Tokyo, Japan). The latter component of the program (wiping with detergents) included wiping of floors with disinfectants (e.g., Dimension II; Butcher, Marlborough, MA, USA) and wiping of other places such as lockers or overbed tables with neutral detergents after wiping with disinfectants (e.g., Dimension II). In all hospitals, there were no differences in cleaning method or frequency between the nurse and patient areas.

### ATP method and evaluation of detection limit

**Figure 2:**
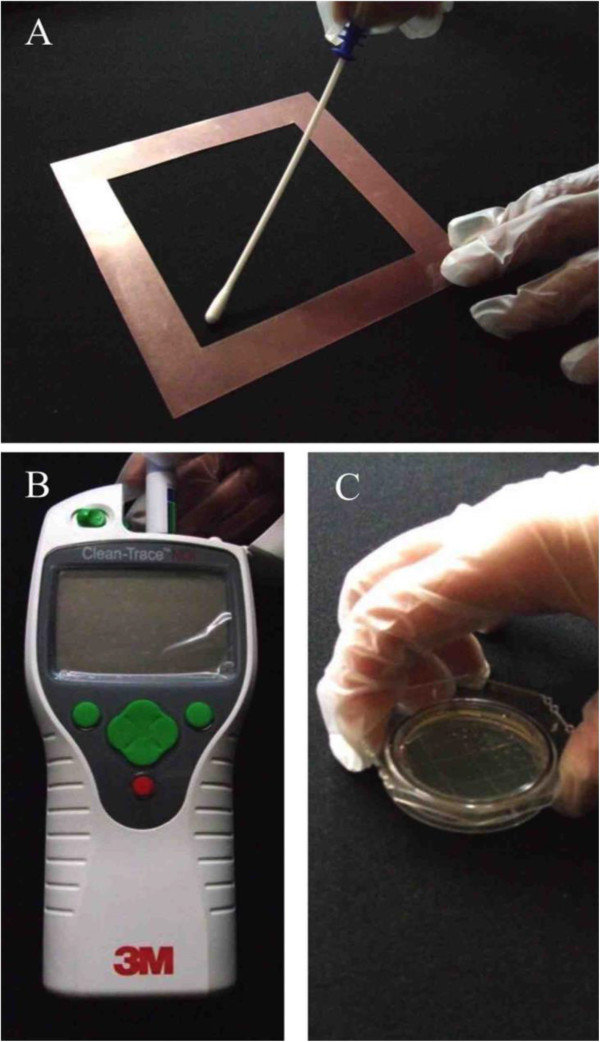
**Sampling images. (A and B)** ATP method. **(C)** Stamp method.

ATP bioluminescence was performed using a 3 M Clean-Trace ATP System (Sumitomo 3 M Limited, Tokyo, Japan) according to the manufacturer’s instructions. Samples were collected by wiping a 10- × 10-cm^2^ area of each surface with a swab supplied with the system, and ATP amounts in the swab were immediately measured in duplicate [Figure 2A (a frame with square for sampling) and B (luminometer)]. The averaged data obtained as bioluminescence relative light units (RLU) were expressed as the fold change over the detection limit value estimated as follows. The detection limit of the ATP method was determined using a wiped sample obtained from a dry plastic dish (10 × 10 cm) placed in a biological safety cabinet that had been spiked with 100 μl of phosphate-buffered saline with defined colony-forming units (CFU) of bacteria (*Escherichia coli*, *Staphylococcus aureus*, and *Bacillus subtilis*). All bacterial strains used for this study were from our laboratory stock collections.

### Stamp method and evaluation of detection limit

Commercial stamp agar based on soybean casein digest (Clean Stamp with 10-cm^2^ surface area; Nissui Pharmaceutical Co., Ltd., Tokyo, Japan) for monitoring environments for microorganism contamination was used for the assay (Figure 2C). The stamp method was performed in parallel with the ATP method. The agar plate was cultured for 5 to 7 days under aerobic conditions with moisture at 30°C, and colonies were counted. The data were estimated as CFU per 10 cm^2^. The stamped agar plates were immediately cultured. The data obtained as CFU were expressed as the fold change over the detection limit estimated as follows. The detection limit of the stamp agar method was examined on randomly chosen student tables (*n* = 19) in a lecture room of our department (Faculty of Health Sciences, Hokkaido University) during winter vacation. The samples used to determine the detection limit were collected from each table before and 2 min after treatment with a neutral detergent (Magiclean; Kao). Data are expressed as CFU per stamp: total of values obtained before and after treatment are used as the detection limit.

### Ethical consideration

The need for ethical approval was waived by the ethical committee of each hospital in this study. Meanwhile, before collecting samples or data, we explained the study design, and informed consent was orally obtained from all medical staff members and hospital inpatients intending to participate in this study. Furthermore, during sample or data collection, we protected the privacy and confidentiality of personal information under supervision of each of the hospital managers and in accordance with the Helsinki Declaration [21].

### Statistical analysis

Comparison of bacterial contamination levels (percentage or relative fold change) was assessed by Student’s *t*-test. Spearman’s correlation index *r* was calculated using statistical analysis software [SPSS Statistics (15.0 J), IBM, Tokyo, Japan]. A *p* value of <0.05 was considered to be statistically significant.

## Results and discussion

### Determination of detection limits for ATP and stamp methods

**Figure 3:**
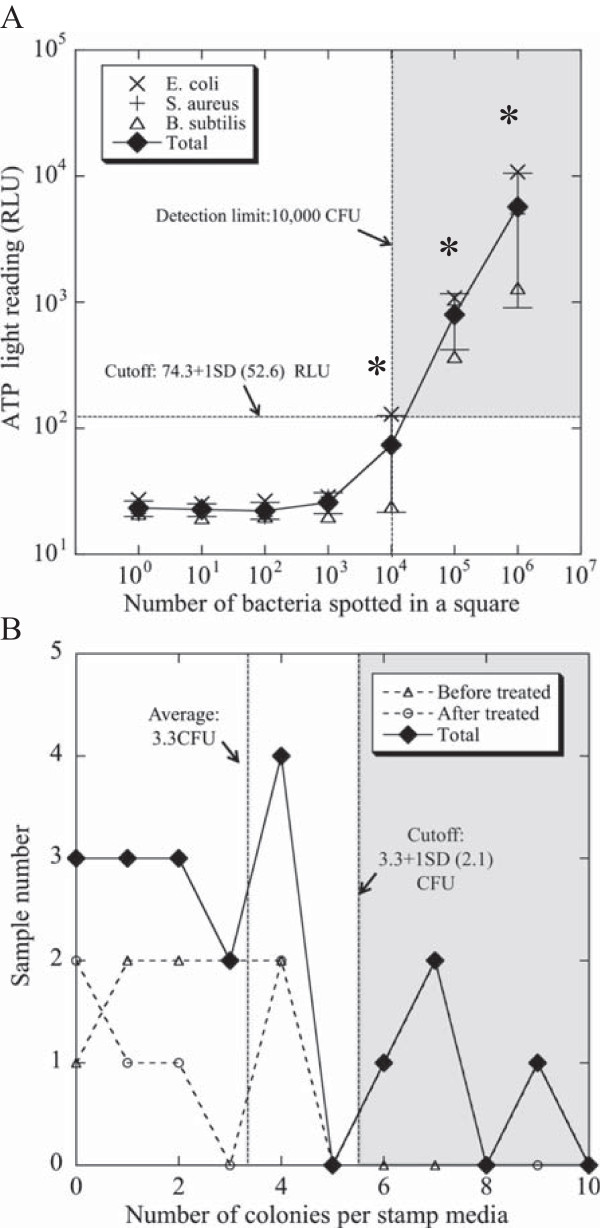
**Assessment of background for ATP and stamp methods. (A)** The background of the ATP method was estimated by a spiked experiment with a diluted bacterial solution of three bacteria (*E. coli*, *S. aureus*, and *B. subtilis*) (10^0^–10^6^ CFU). The background value was 74.3 + 1 standard deviation (SD) (52.6) RUL, equivalent to 10,000 CFU. The experiments were independently performed at least three times. The data show the mean ± SD. *p < 0.05 vs. the value of the sample spiked with 10^0^ CFU (Student’s *t* test). **(B)** The background of the stamp agar method was randomly examined on student tables (*n* = 19) in a lecture room of our department (Faculty of Health Sciences, Hokkaido University) during winter vacation, and the total CFU detected before and after treatment with a neutral detergent was determined as the detection limit of the stamp method (See Methods). The detection limit was estimated as 3.3 + 1 SD (2.1) CFU.

The detection limits of the ATP and stamp methods have clearly differed from each other in all previous studies on this topic (see review, Reference [22]), although each of the average benchmarks was 100 RLU per 100 cm^2^ of ATP and <25 CFU per 10 cm^2^ of microbial colonies. Therefore, we originally evaluated the detection limits of the ATP and stamp methods. As a result, the detection limit of the ATP method by the spiked experiment with *Escherichia coli*, *Staphylococcus aureus*, and *Bacillus subtilis* was estimated at 127 [74.3 (average) + 52.6 (standard deviation: SD)] RLU per 100 cm^2^ (Figure 3A). There were no statistically significant differences among the three bacterial species. We also evaluated the detection limit of the stamp method by taking samples from student tables (*n* = 19) in a lecture room of our department during vacation. The detection limit of the stamp method was defined as 5.3 [3.3 (average) + 2.1 (SD)] CFU per 10 cm^2^ (see Figure 3B, “total”).

We found that the ATP values increased when the spiked bacterial numbers were more than 10,000 CFU, indicating that the bacterial detection limit of the ATP method was very low. This is not surprising because a trait of the ATP method is detection of residual ATP, not only microbial contamination, in “high-touch sites” as an indicator of general organic contamination [11-13]. Moreover, it has been noted that the use of the ATP method to rapidly monitor hospital cleanliness clearly increased the motivation of domestic staff or housekeepers via education and monitoring with feedback, demonstrating an indirect connection with the reduction of key environmental organisms [23,24]. Therefore, the ATP method has become the most popular method available for monitoring hospital cleanliness [11-15].

We also confirmed that the higher sensitivity of the stamp method is an advantage over the ATP method in terms of precisely monitoring microbiologic contamination of hospitals. Although the stamp culture could not identify all organisms (for instance, it was not possible to identify anaerobic microorganisms), the aerobic culture condition that we used could identify most microorganisms related to hospital infection. Therefore, the stamp method is an appropriate and effective monitoring system for evaluating microbiological contamination associated with high-touch sites of hospitals, allowing for direct detection of hospital-acquired pathogens. Thus, because monitoring with a combination of the ATP and stamp methods could provide advantages in terms of covering a wide range of potential threats to adequate hospital cleanliness, both methods were used in this study.

### Positive frequencies of the ATP and stamp methods in hospital environments

**Figure 4:**
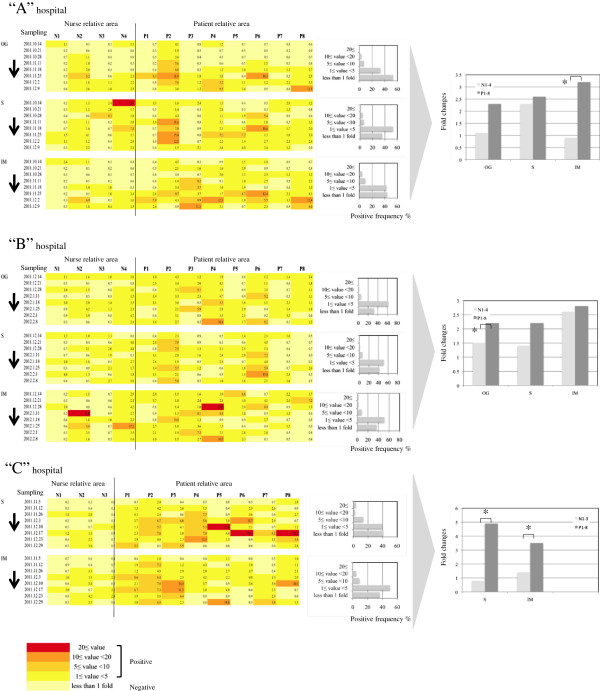
**Comparison of positive frequencies estimated by the ATP method in hospital environments.** (Left panels) Positive frequencies and relative fold change values estimated by the ATP method. Color changes show the degree of ATP values, ranging from highest contamination (red) to lowest contamination (yellow). The numbers in each of the squares show fold change values. **“A”** hospital, >500 beds with the following sampling period, October 2011 to December 2011. **“B”** hospital, 100–500 beds with the following sampling period, December 2011 to February 2012. **“C”** hospital, <100 beds with the following sampling period, November 2011 to December 2011. N1–3 or 4, nurse areas. P1–8, patient areas. OG, Department of Obstetrics and Gynecology. S, Department of Surgery. IM, Department of Internal Medicine. Arrows indicate the order of the sampling times. (Middle panels) Distribution of positive frequencies in each unit of each hospital. Five categories were provided (20 ≤ value, 10 ≤ value < 20, 5 ≤ value < 10, 1 ≤ value < 5, and <1-fold). (Right panels) Comparison of nurse and patient areas with the ATP method. The data show the relative fold change over the detection limit of the ATP method. *p < 0.05 (Student’s *t*-test).

**Figure 5:**
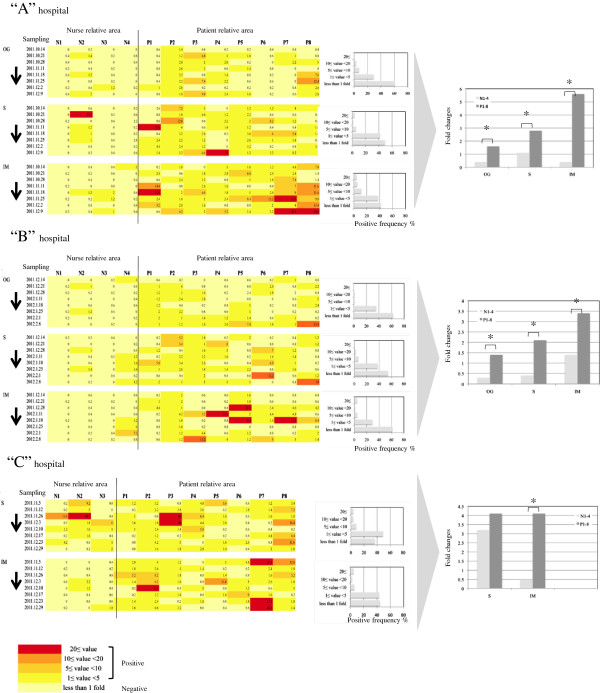
**Comparison of positive frequencies estimated by the stamp method in hospital environments.** (Left panels) Positive frequencies and relative fold change values estimated by the stamp method. Color changes show the degree of the stamp value, ranging from highest contamination (red) to lowest contamination (yellow). The numbers in each of the squares show fold change values. **“A”** hospital, >500 beds with the following sampling period, October 2011 to December 2011. **“B”** hospital, 100–500 beds with the following sampling period, December 2011 to February 2012. **“C”** hospital, <100 beds with the following sampling period, November 2011 to December 2011. N1–3 or 4, nurse areas. P1–8, patient areas. OG, Department of Obstetrics and Gynecology. S, Department of Surgery. IM, Department of Internal Medicine. Arrows indicate the order of the sampling times. (Middle panels) Distribution of positive frequencies in each unit of each hospital. Five categories were provided (20 ≤ value, 10 ≤ value < 20, 5 ≤ value < 10, 1 ≤ value < 5, and <1-fold). (Right panels) Comparison of nurse and patient areas with the stamp method. The data show the relative fold change over the detection limit of the stamp method. *p < 0.05 (Student’s *t*-test).

The positive rates of both the ATP and stamp methods, obtained through time-course monitoring in hospital environments, were evaluated [Figure 4 (ATP method), Figure 5 (stamp method)]. As a result, a significant difference was seen between the positive frequencies (fold change of >1) of the ATP and stamp methods (Figures 4 and 5, left panels) [average positive frequency per hospital: 57.71% (ATP method) vs. 42.58% (stamp method), *p* = 0.016]. The ATP values showed the presence of remnant organic matter spread over a wide range of hospital environments via hand touching despite the fact that adequate hospital cleanliness by visual assessment with usual wiping or cleaning had been executed in each of the hospitals. In fact, the finding that the ATP method captured a wider range of sites (fold change of >1; 450 sites) than the stamp method (fold change of >1; 359 sites) supports the fact that the ATP method is capable of covering high-touch sites, possibly including those with bacterial contamination. Meanwhile, the stamp method results indicated potential sites of microbial contamination in the hospitals that were not estimated by the ATP method; the number of maximum fold changes (≥20 per hospital) estimated by the stamp method was significantly higher than that estimated by the ATP method [average numbers ± SD per hospital: 2 ± 1 (ATP method) vs. 6 ± 1 (stamp method), *p* = 0.008] (Figures 4 and 5, middle panels). It is likely that the sites were unevenly distributed, mostly concentrating on patients’ rooms on particular windowsills or overbed tables (see Figure 5, column P4-8), suggesting that these areas received inadequate cleaning.

Cleaning a crowded public area of a ward with isolation rooms containing patients is reportedly far more complex than cleaning offices such as nurse stations [22,25]. To confirm this, we compared nurse (N1–3 or N1–4) and patient areas (P1–8) in each of the hospital units, hypothesizing that nurse areas were cleaner than patient areas because patient areas contain more medical personnel and inpatients. As expected, a significant difference between nurse and patient areas was found using the stamp method (8 units, *p* < 0.05) and the ATP method (4 units, *p* < 0.05) (Figures 4 and 5, right panels). These results indicate that maintaining hospital cleanliness of inpatient areas with more complicated medical personnel is difficult, supported by previous study [20].

Interestingly, the data of both the ATP and stamp methods demonstrated considerable variability, regardless of daily visual assessment with usual wiping or cleaning. In addition, positive surfaces were often but irregularly seen, although the exact reason why hospital contamination by organic matter or microbes irregularly occurred remains unknown. Furthermore, guidelines emphasize the importance of hospital cleanliness [8-10], but give little practical advice on how to achieve this. Medical staff members, including nurses, may be too busy to properly clean furniture and equipment, and medical care support is considered to be of higher priority than wiping or cleaning the tops of lockers or overbed tables. Medical staff members’ workloads may reach critical limits in Japanese hospitals, as well as those in other developed countries [22,25]. Antimicrobial coatings containing heavy metals or biocides are currently available for items such as clothes, linen (sheets and curtains), furniture (lockers and overbed tables), and high-touch sites [26-28]. These new, innovative products could be expected to successfully achieve appropriate hospital cleanliness under the present medical and housekeeping activity levels.

**Figure 6:**
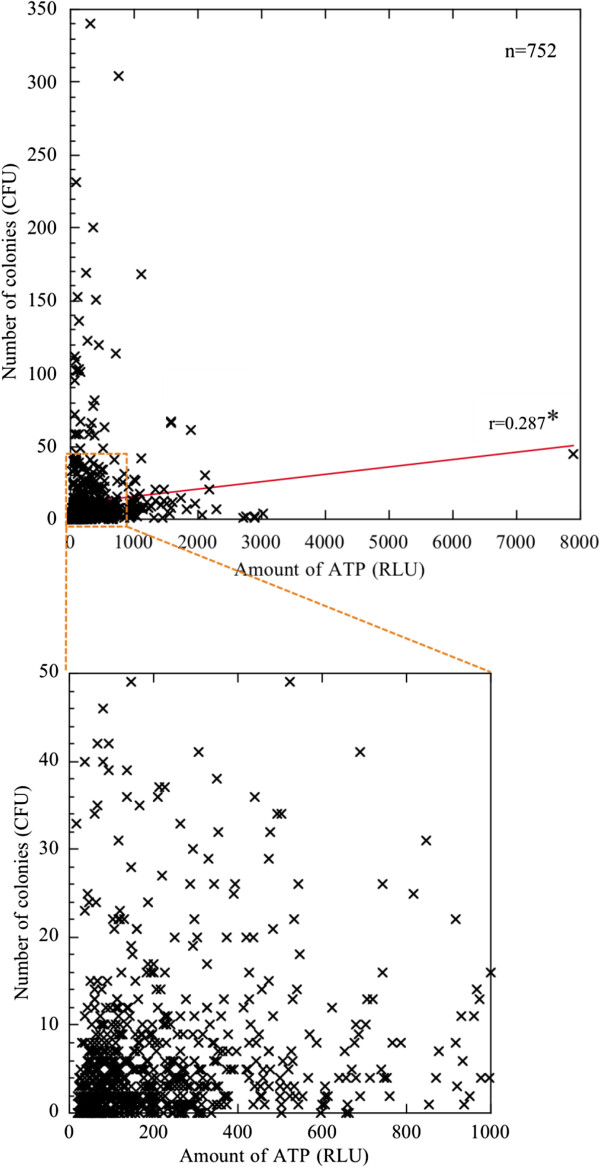
**Correlation between ATP values and stamp values estimated on all 752 hospital surfaces.** The x axis shows the amount of ATP (RLU) estimated by the ATP method, and the y axis shows the CFU estimated by the stamp method. The red line is the correlation line. The small dashed square is enlarged in the lower panel. *r*, Spearman’s correlation index. *p < 0.05 (showing minimal statistical significance).

As expected, the correlation between the values obtained from the ATP and stamp methods was limited (*r* = 0.287) and had minimal statistical significance (Figure 6). Because the rate of both methods being positive was 28.3% (data not shown), it is possible that this small overlap could be responsible for the weak correlation. Several studies have reported a poor correlation between ATP and stamp evaluations in hospital environments [29,30], which is consistent with our results, highlighting a large degree of contaminated hospital areas. In addition, to determine whether medical staff member or inpatient numbers correlated with ATP and stamp values, we calculated the correlation between ATP or stamp values and the number of either personnel or hospital inpatients. None of the correlations were statistically significant (Table 1), strongly suggesting the presence of some unknown other factor related to actual hospital contamination in Japan hospitals.

**Table 1:** Correlation index between either ATP or stamp values and number of either staffs or hospital inpatients

	**ATP method**			**Stamp method**		
	**Inpatients**	**Medical staffs**	**Total**	**Inpatients**	**Medical staffs**	**Total**
Pearson’s correlation index	−0.05	−0.022	−0.39	−0.034	−0.07	−0.036
*p* values*	0.169	0.551	0.29	0.352	0.855	0.321

*less than 0.05 was considered significant.

Although cleaning within hospitals is a major budget item, there are currently few accurate data with which to judge cleaning efficacy, as mentioned above. Our results caution against the popular belief that regular visual assessment with wiping is sufficient for maintaining hospital cleanliness in Japan hospitals with a tendency toward long-term care.

## Conclusion

The visualized cleanliness of Japanese hospitals showed considerable variability, suggesting insufficient ongoing daily cleaning. Based on certain monitoring parameters of hospital cleanliness using the ATP or stamp method, further cleaning tasks or definite role sharing for medical staff members and housekeepers, in conjunction with new techniques such as material coatings, should be considered to maintain adequate hospital environments in facilities with a tendency towards long-term care, such as those in Japan.

## Competing interests

The authors declare that they have no competing interests.

## Authors’ contributions

RW, TS, and RY performed the sampling and the assessment with the ATP and stamp methods. JM, SN, and YH performed technical assistance in culturing and equipment management. RW, TS, RY, and HY conducted the statistical analysis. RW, TS, RY, and HY designed the study. RY and HY supervised the practical work and data management. HY wrote the manuscript. All authors approved the final version of the manuscript.
